# Rational design of high-quality 2D/3D perovskite heterostructure crystals for record-performance polarization-sensitive photodetection

**DOI:** 10.1093/nsr/nwab044

**Published:** 2021-03-16

**Authors:** Xinyuan Zhang, Lina Li, Chengmin Ji, Xitao Liu, Qing Li, Kun Zhang, Yu Peng, Maochun Hong, Junhua Luo

**Affiliations:** State Key Laboratory of Structural Chemistry, Fujian Institute of Research on the Structure of Matter, Chinese Academy of Sciences, Fuzhou 350002, China; University of Chinese Academy of Sciences, Beijing 100049, China; State Key Laboratory of Structural Chemistry, Fujian Institute of Research on the Structure of Matter, Chinese Academy of Sciences, Fuzhou 350002, China; University of Chinese Academy of Sciences, Beijing 100049, China; State Key Laboratory of Structural Chemistry, Fujian Institute of Research on the Structure of Matter, Chinese Academy of Sciences, Fuzhou 350002, China; University of Chinese Academy of Sciences, Beijing 100049, China; State Key Laboratory of Structural Chemistry, Fujian Institute of Research on the Structure of Matter, Chinese Academy of Sciences, Fuzhou 350002, China; University of Chinese Academy of Sciences, Beijing 100049, China; University of Chinese Academy of Sciences, Beijing 100049, China; Hangzhou Institute for Advanced Study, University of Chinese Academy of Sciences, Hangzhou 310024, China; School of Physical Science and Technology, ShanghaiTech University, Shanghai 201210, China; State Key Laboratory of Structural Chemistry, Fujian Institute of Research on the Structure of Matter, Chinese Academy of Sciences, Fuzhou 350002, China; State Key Laboratory of Structural Chemistry, Fujian Institute of Research on the Structure of Matter, Chinese Academy of Sciences, Fuzhou 350002, China; University of Chinese Academy of Sciences, Beijing 100049, China; State Key Laboratory of Structural Chemistry, Fujian Institute of Research on the Structure of Matter, Chinese Academy of Sciences, Fuzhou 350002, China; School of Chemistry and Chemical Engineering, Jiangxi Normal University, Nanchang 330022, China; University of Chinese Academy of Sciences, Beijing 100049, China

**Keywords:** polarization-sensitive photodetection, hybrid perovskite, single-crystalline heterostructure, high polarization sensitivity

## Abstract

Polarization-sensitive photodetection is central to optics applications and has been successfully demonstrated in photodetectors of two-dimensional (2D) materials, such as layered hybrid perovskites; however, achieving high polarization sensitivity in such a photodetector remains extremely challenging. Here, for the first time, we demonstrate a high-performance polarization-sensitive photodetector using single-crystalline 2D/3D perovskite heterostructure, namely, (4-AMP)(MA)_2_Pb_3_Br_10_/MAPbBr_3_ (MA = methylammonium; 4-AMP = 4-(aminomethyl)piperidinium), which exhibits ultrahigh polarization sensitivity up to 17.6 under self-driven mode. To our knowledge, such a high polarization selectivity has surpassed all of the reported perovskite-based devices, and is comparable to, or even better than, the traditional inorganic heterostructure-based photodetectors. Further studies reveal that the built-in electric field formed at the junction can spatially separate the photogenerated electrons and holes, reducing their recombination rate and thus enhancing the performance for polarization-sensitive photodetection. This work provides a new source of polarization-sensitive materials and insights into designing novel optoelectronic devices.

## Teaser

High-quality 2D/3D perovskite heterostructure crystals have been successfully created to demonstrate a self-driven polarization-sensitive photodetector, which achieves a record-high polarization sensitivity up to 17.6.

## INTRODUCTION

Polarization-sensitive photodetectors, based on anisotropic semiconductors, have exhibited overwhelming advantages in specialized applications, including astronomy, remote sensing and polarization-division multiplexing [1–4]. For the active layer of polarization-sensitive photodetectors, primary research focuses on one-dimensional (1D) nanowires or nanotubes, such as InP [5], ZnO [6] and BaTiS_3_ [7] due to their geometric anisotropy. Although these 1D materials can achieve high polarization sensitivity (*I*_max_/I_min_) of up to ∼10, the complicated patterning and aligning process limit their diversified application [8,9]. For this reason, 2D materials, including black phosphorus (BP) [10], GeSe_2_ [11] and ReS_2_ [12], with their inherently low-symmetry individual layers and attractive *ab*-plane anisotropy, have been widely investigated for polarization-sensitive photodetection and enjoyed great success [13]. Nevertheless, the optic axis of those layered materials is generally the *c*-axis and it is difficult to access their *ac*-plane with large anisotropy.

Recently, strong polarization sensitivity has also been demonstrated in 2D organic-inorganic hybrid perovskites, where inorganic slabs and organic spacers are alternatively arranged in parallel layered structures [14,15]. These atomic-scale layers running along a high-symmetry principal axis create a quantum-confined motif, which endows natural anisotropy for 2D hybrid perovskites. More importantly, compared with inorganic 2D materials, the solution accessibility of hybrid perovskites makes it possible to obtain their large crystals at low cost, offering exciting opportunities to incorporate crystal out-of-plane anisotropy for polarization-sensitive photodetection. So far, numerous polarization-sensitive photodetectors of 2D hybrid perovskites have been reported, fully revealing their promise in this field [16,17]. However, limited by the absorption anisotropy of the material structure, polarization sensitivity of such a device remains modest (∼2). Researchers tried to solve this problem by developing 2D perovskite-type ferroelectrics, but they have very limited selection [18]. Thus, a new strategy to design 2D hybrid perovskites with large, easily accessible anisotropy for polarization-sensitive photodetection is urgently desired.

Heterostructures, which engineer physical properties while retaining the intrinsic nature of each component, provide a clue to address this challenge [19–24]. Intuitively, construction of heterostructures can enhance the optical absorption and free-carrier densities of the composite. Furthermore, the built-in electric field at the heterostructure interface can spatially separate the photogenerated electron-hole pairs, significantly reducing the recombination rate and thereby improving the sensitivity for polarization-sensitive photodetectors. A typical example is that a photodetector using BP/MoS_2_ heterostructure achieved a high polarization sensitivity up to 22, in striking contrast with that of pure BP (3.5) [25]. Therefore, constructing single-crystalline heterostructures of anisotropic 2D hybrid perovskites would be a promising way to realize devices with high polarization sensitivity.

Here, a single-crystalline 2D/3D hybrid perovskite heterostructure, namely (4-AMP)(MA)_2_Pb_3_Br_10_/MAPbBr_3_ (MA = methylammonium; 4-AMP = 4-(aminomethyl)piperidinium), is created to achieve high-performance polarization-sensitive photodetectors. Although previous studies have demonstrated photodetectors using such a heterostructure [26], here we make a significant breakthrough, showing that the device is polarization-sensitive and can be competitive with excellent inorganic polarization-sensitive photodetectors. The built-in electric field at the junction enables efficient carrier transport/separation, greatly contributing to the polarization-sensitive activity. Under zero bias, polarization sensitivity of the heterostructure-based detector can reach a remarkable high of 17.6. This innovative study broadens the choice of materials that can be used for high-performance polarization-sensitive photodetectors, and correspondingly, the design strategies.

## RESULTS AND DISCUSSION

The 2D/3D (4-AMP)(MA)_2_Pb_3_Br_10_/MAPbBr_3_ heterostructure is formed by vertically integrating (4-AMP)(MA)_2_Pb_3_Br_10_ and MAPbBr_3_ perovskites via organic spacer as shown in Supplementary Fig. 1. Corresponding bulk crystals are successfully obtained through a temperature-cooling solution method (see Supplementary Discussion 1 and Supplementary Fig. 2) [26] and feature high crystallinity and sharp interfaces (see Supplementary Discussion 2 and Supplementary Figs 3–5). Ultraviolent-visible absorption measurement supports the formation of the 2D/3D heterostructure and reveals a significantly improved optical absorption after 3D MAPbBr_3_ integration (Supplementary Fig. 6).

**Figure 1.**
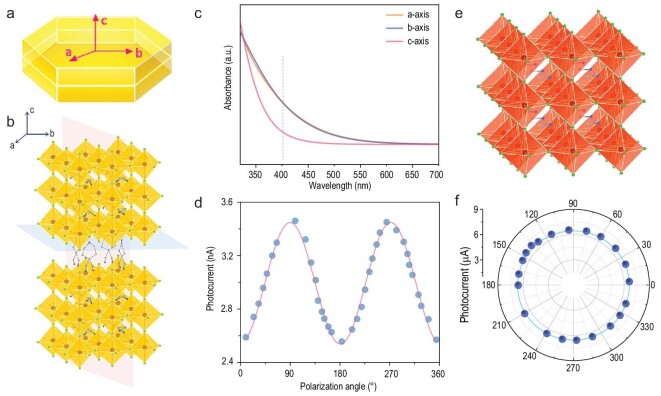
Anisotropy of (4-AMP)(MA)_2_Pb_3_Br_10_ and its 3D counterpart. (a) Growth morphology for (4-AMP)(MA)_2_Pb_3_Br_10_ crystals. (b) Structure of (4-AMP)(MA)_2_Pb_3_Br_10_ along different lattice plane. (c) Calculated absorbance along different axes of (4-AMP)(MA)_2_Pb_3_Br_10_. (d) Photocurrent for (4-AMP)(MA)_2_Pb_3_Br_10_-based polarization-sensitive photodetector as a function of polarized light incident angle under 10 V bias at 405 nm. The *c*-axis direction is defined as 0° polarization. (e) Structure of 3D MAPbBr_3_. (f) Polar plot of the angle-resolved photocurrent as a function of polarization angle of MAPbBr_3_ measured at 405 nm under 10 V bias.

Before studying the polarization-sensitive behavior of the 2D/3D heterostructure, we first measure the crystal structure, along with anisotropic optical absorption of (4-AMP)(MA)_2_Pb_3_Br_10_ to characterize its anisotropy. The basic building blocks of the inorganic framework in (4-AMP)(MA)_2_Pb_3_Br_10_ are PbBr_6_ octahedra, which are mutually connected via corner-sharing forming [PbBr_6_]^4–^ layers along the *ab*-plane and are separated by 4-AMP^2+^ diamine along the *c*-axis, thus forming a 2D structure with two inequivalent directions within the out-of-plane lattice (Fig. 1a and b). This structural anisotropy fully leads to the absorption anisotropy of (4-AMP)(MA)_2_Pb_3_Br_10_ along three crystallographic axes, of which the approximate ratio of absorbing intensity (*α_b_/α_c_*) at 405 nm is ∼1.9 (Fig. 1c). The corresponding photodetector based on (4-AMP)(MA)_2_Pb_3_Br_10_ crystal shows a polarization sensitivity (*I*_max_/*I*_min_, where *I*_max_ and *I*_min_ are photocurrent maximum and minimum when light is polarized parallel to the *b*-axis and *c*-axis, respectively) of ∼1.5 (Fig. 1d), which is comparable to that of many other reported 2D anisotropic materials such as (BA)_2_MAPb_2_Br_7_ (BA = *n*-butylammonium) (Supplementary Table 1) [14]. In contrast, the photocurrent of the MAPbBr_3_-based device exhibits a stable size with the transformation of the polarization angle, directly due to the isotropic crystal structure of MAPbBr_3_ (Fig. 1e and f).

**Figure 2.**
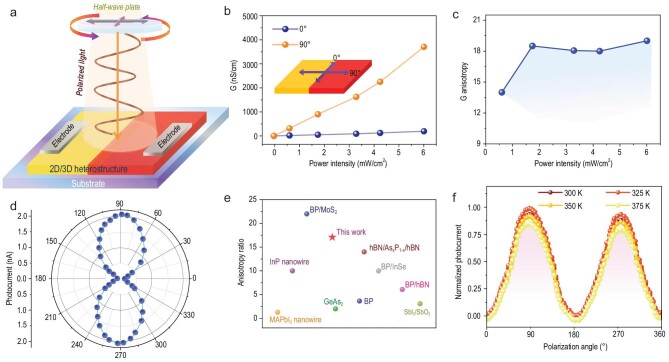
Device performance of heterostructure-based polarization-sensitive photodetector. (a) Schematic structure of the polarization-sensitive photodetector based on the 2D/3D heterostructure crystal. (b) Photoconductivity parallel and perpendicular to the heterostructure interface. (c) Photoconductivity anisotropy versus excitation power. (d) Angle-resolved photocurrent as a function of polarization angle measured at 405 nm under zero bias. (e) Experimental polarization sensitivities of some reported polarized light detectors. (f) Angle-dependent photocurrent of the present device measured at different temperatures.

Then, we leverage the anisotropy of the 2D perovskite to develop a polarization-sensitive photodetector based on the 2D/3D heterostructure. As schematically illustrated in Fig. 2a, a two-terminal photodetector is fabricated on the heterostructure crystal. A thin layer of Ag is coated on MAPbBr_3_ perovskite as the cathode, and the corresponding (4-AMP)(MA)_2_Pb_3_Br_10_ perovskite is the anode. The large crystal size and regular crystal shape allow for easy device fabrication. As shown in Supplementary Fig. 7, the expected rectifying behavior is observed in the dark, arising from the built-in electric field formed at the heterostructure interface [27,28]. Upon illumination, the heterostructures exhibit excellent photocurrent generation

 

characteristics and an obvious photovoltaic effect (Supplementary Fig. 8). This distinct photovoltaic effect can enable the present device to function as a self-driven photodetector that operates without external bias. Such an excellent performance from a heterostructure photodetector encourages us to study its polarization-sensitive detection. The incident polarization light (405 nm) is controlled by a polarizer and half-wave plate; then illuminates at the heterostructure interface where the horizontal direction is defined as 0° polarization (Fig. 2a). During the experiment, 0 V bias is applied between the electrodes, thus the photoresponse is solely driven by the built-in electric field. Figure 2b illustrates the photoconductivity at the junction area as a function of laser intensity and polarization. In general, conductance of the 2D/3D heterostructure increases by two to three orders of magnitude as excitation power intensity increases. Notably, the photoconductivity exhibits an obvious anisotropy with 0° excitation producing G that is over one order of magnitude lower than 90° excitation (Fig. 2c). When continuously rotating the excitation polarization vector from 0° to 360°, the polarization photoresponse changes periodically (Fig. 2d).

**Figure 3.**
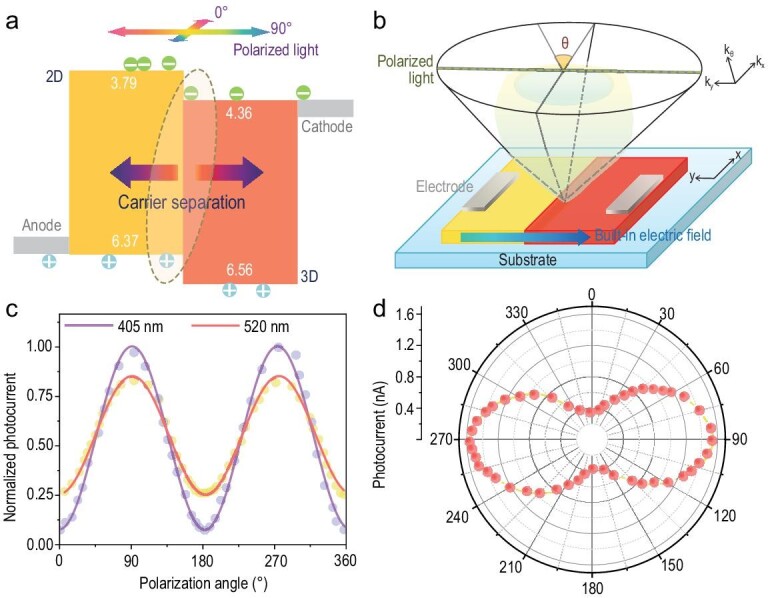
Mechanism of the polarization-sensitive photodetector. (a) Band alignment of the 2D/3D heterostructure. (b) The schematic shows how the linearly polarized light is aligned with the direction of the built-in electric field. (c) Angle-resolved photocurrent of the heterostructure photodetector measured at 405 and 520 nm. (d) Polar plots of photocurrent for the heterostructure device measured under 520 nm.

In striking contrast to the modest polarization sensitivity in the (4-AMP)(MA)_2_Pb_3_Br_10_-based device, the measured polarization sensitivity of the heterostructure polarization-sensitive photodetector can reach a remarkable high of 17.6. To the best of our knowledge, this is the highest reported value among perovskite-based devices, and is comparable to or even better than that of widely studied inorganic heterostructures, such as BP/MoS_2_ (∼22) [25], hBN/(b-As_x_P_1-x_)/hBN (∼14) [29] and BP/InSe (∼10.76) [21]. Experimental polarization ratios of some reported polarized light photodetectors are summarized in Supplementary Table 1 and Fig. 2e. The polarization sensitivity of our device shows no significant changes as a function of temperature, indicating its favorable thermal stability (Fig. 2f). Such an enhanced polarization sensitivity can be ascribed to the following aspects: (i) high crystalline quality of the grown heterostructure crystals—the low amounts of boundary-induced defects and chemical disorders render our heterostructure an ideal medium for charge transport [30,31]; (ii) the ultralow dark current—the low dark current of the heterostructure device at zero bias is beneficial in improving the polarization sensitivity [32]; (iii) more importantly, the existence of a built-in electric field [2,25]. We extract exciton binding energy (*E_b_*) of (4-AMP)(MA)_2_Pb_3_Br_10_ from its temperature-dependent photoluminescence data (Supplementary Fig. 9). The *E_b_* is estimated to be 160 meV, much higher than that reported in MAPbBr_3_ (5–25 meV) [33]. This large exciton binding energy requires high energy to separate those light-generated Frenkel excitons and causes a relatively high recombination rate in 2D materials, which makes it difficult to achieve sensitive polarized photodetection. According to the ultraviolet photoelectron spectroscopy (UPS) analysis (Supplementary Fig. 10), our 2D/3D heterostructure adopts a type-II band alignment. This alignment favors photogenerated carrier separation [34–37]. Driven by the built-in electric field, electrons move to the 3D perovskite while holes move to the 2D perovskite (Fig. 3a). The direct result of this is that the recombination probability for electrons and holes during their motion to opposite electrodes will be greatly reduced.

Within the heterostructure, the angular distribution of momentum of hot carriers excited by linearly polarized light along y direction is depicted in Fig. 3b and can be expressed as [38,39]:

$$\begin{equation*}
{\left| \psi \right|^2} \propto {A^2} + {B^2} - 2AB{\rm cos}2\theta ,
\end{equation*}$$

where *A* and *B* represent the overlap integral between wave functions of electron and hole. Based on the equation, the momentum of hot carriers under polarization excitation should exhibit significant directionality [40,41]. When the polarized light is parallel to the built-in electric field (incident along y direction), the photogenerated carriers have maximum momentum in the direction of built-in electric field, which can effectively separate those generated electron-hole pairs, reducing their recombination and thus leading to pronounced *I*_max_. With polarized light gradually deviating from the direction of the built-in electric field, the momentum of photogenerated carriers gradually decreases and reaches a minimum when perpendicular to the built-in electric field. The exciton separation/transport becomes weak, resulting in ultralow *I*_min_. Therefore, the polarization sensitivity of our heterostructure is greatly amplified compared to pure 2D perovskites. To further understand this issue, we measured the polarization sensitivity of the heterostructure detector with incident energy lower than the 2D component bandgap. At a wavelength of 520 nm, the polarization sensitivity of the device is estimated to be 9.2, lower than that measured at 405 nm (Fig. 3c and d). Generally, for incident photons with energy larger than the bandgap of the two phases (λ < 470 nm), the light will be absorbed by both (4-AMP)(MA)_2_Pb_3_Br_10_ and MAPbBr_3_, and the excited electron-hole pairs will be quickly separated by the built-in electric field, giving rise to pronounced photocurrent. When the photon energy for incident light is between their bandgaps (470 nm < λ < 570 nm), photoexcited carriers would mainly be generated in the MAPbBr_3_, then overcome the contact barrier and move to the (4-AMP)(MA)_2_Pb_3_Br_10_. Hence the electron-hole pairs become less efficient in generation/separation and tend to recombine instead, which causes lower polarization sensitivity [21]. Therefore, we conclude that the built-in electric field may be the dominant factor that influences the polarization sensitivity of our heterostructure.

**Figure 4.**
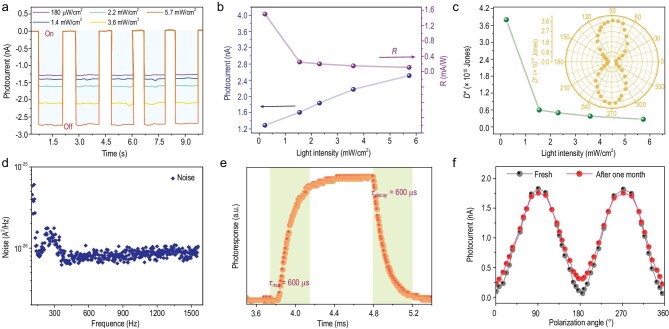
Performance and stability of the heterostructure polarization-sensitive photodetector. (a) Polarized photocurrent of the device measured with different light intensities. (b) Incident-light power dependence of photoresponsivity and photocurrent of the heterostructure. (c) Incident-light power dependence of detectivity of the device. The inset is the polarization dependence of D^*^ as a function of polarization angle provided in the polar plot. (d) The current noise power spectra at 0 V bias. (e) The response time of the heterostructure polarization-sensitive photodetector at 405 nm. (f) Angle-resolved photocurrent of the heterostructure detector after storage in air for 30 days.

Figure 4a depicts the temporal response of the device with different irradiation powers (at 0 V bias, λ = 405 nm). With an ultralow dark current of 8 × 10^–6^ nA, the important parameter—on/off ratio of photocurrent to dark current—can be ∼10^5^ under the given conditions, exceeding the majority of reported detectors based on hybrid perovskites (Supplementary Table 1). To quantitatively evaluate the performance of polarization-sensitive photodetectors, we extract the responsivity ($R$), that is, the sensitivity to incident light. *R* can be derived by $R\ = \ \frac{{{J_{ph}}}}{{{I_{\rm {light}}}}}$, in which *J_ph_* represents the photocurrent density and ${I_{{\rm light}}}$ is incident light intensity. As shown in Fig. 4b and Supplementary Fig. 11, *R* increases sharply with the decreased illuminated intensity, owing to the increased photoconductive gain. Peak *R* of 1.5 mA/W is achieved under 180 μW/cm^2^ light illumination. *R* of the devices based on standalone 2D and 3D components is also provided in Supplementary Fig. 12 for comparison. Further, for a photodetector operated at 0 V bias, the specific detectivity (D^*^) can be defined as [25]:

$$\begin{equation*}
{\rm{\ }}\ {D^*} = \ R{\left( {\frac{{4kT}}{{{R_0}A}} + 2q{\emptyset _b}\frac{{Rhc}}{\lambda }} \right)^{ - \frac{1}{2}}},
\end{equation*}$$

where *R*_0_ is the zero-bias resistance, *A* is the illuminated area of the photodetector, *q* is the elementary charge, ${\emptyset _b}$ is the background flux density, *λ* is the wavelength, *c* is the speed of light in a vacuum, *T* is the detector temperature, and *h* and *k* are the Planck and Boltzmann constants. The second term $2q{\emptyset _b}\frac{{Rhc}}{\lambda }$ accounts for contributions to noise from fluctuations in the thermal background, which are negligible in our case, as $\frac{{4kT}}{{{R_0}A}} > 2q{\emptyset _b}\frac{{Rhc}}{\lambda }$. Thus, the D^*^ can be calculated by the formula: $\ {D^*} = \ R{( {\frac{{4kT}}{{{R_0}A}}} )^{ - \frac{1}{2}}}$. Figure 4c shows the D^*^ extracted from this approach at room temperature. The highest value of 3.8 × 10^10^ Jones is comparable to many other state-of-the-art detectors based on integrated inorganic heterostructures (Supplementary Table 1). Improvements in D^*^ can be achieved by decreasing the heterostructure bulk trap concentration during crystal growth and reducing the electronic noise. The polarization dependence of D^*^ as a function of polarization angle is provided in the polar plot of Fig. 4c insertion. That the D^*^ for 90° excitation light is substantially higher than for 0° excitation light is in alignment with the photoresponse in Fig. 2d. Figure 4d shows the measured noise current spectrum of the present heterostructure-based photodetector. The measured low-frequency noise has a value of 1 × 10^–13^ A/Hz^1/2^, which is similar to that of previously reported photodetectors [25,42,43]. Supplementary Fig. 13 demonstrates that the device can work with remarkable reproducibility after a long-term repetition of polarized light ‘on/off’ (∼10^4^ cycles) without baseline drift, suggesting the high reproducibility of our single-crystal device. Based on the definition of response speed, the rise/fall time (*τ*_rise_/*τ*_fall_) is determined to be ∼600/600 μs (Fig. 4e). Moreover, we also measure the powder x-ray diffraction (PXRD) pattern and electrical stability of our heterostructure-based device after it was stored in ambient for 30 days. As illustrated in Supplementary Fig. 14 and Fig. 4f, the characterizations suggest negligible degradation after storage, showing its significant environmental stability. The above results clearly demonstrate the advantages of our 2D/3D heterostructure device in high-performance polarization-sensitive photodetection, which will shed light on new properties for their future application.

## CONCLUSION

In summary, high polarization sensitivity is successfully achieved in a self-driven polarization-sensitive photodetector based on a single-crystalline 2D/3D hybrid perovskite heterostructure that is grown via a delicate solution method. Further studies indicate that the built-in electric field at the junction provides a desirable driving force for carrier transport/separation, mainly accounting for this remarkable polarization sensitivity. Though far from optimized, our observations provide the first demonstration that construction of a 2D/3D perovskite heterostructure can play a significant role in performance enhancement in polarization-sensitive photodetection. We believe that this work will shed light on designing novel optoelectronic devices beyond conventional materials and approaches.

## METHODS

### Materials

The 4-(aminomethyl)piperidinium (4-AMP, 98%), 40% methylamine solution, 48% aqueous hydrobromic acid (HBr) solution and lead bromide (PbBr_2_, 99.9%) were purchased from Aladdin (Shanghai, China). 4-AMPBr_2_ (or MABr) was synthesized by slowly mixing 4-AMP 2 g (or MABr 8 mL) with HBr (10 mL) under continuous stirring at 0°C for 1.5 h. Then, 4-AMPBr_2_ (or MABr) was crystallized from a rotary evaporator at 70°C for 5 h, washed in diethyl ether three times and collected by filtering.

### Device preparation and measurement

Single-crystal photodetectors for both unpolarized- or polarized light detection were fabricated using heterostructure crystals with dimensions of 4 × 5 × 1.2 mm^3^. The electrodes typically had dimensions of 0.5 × 1 mm^2^ laterally and a thickness of ∼0.05 mm. The electrode materials were proven not to have any obvious influence on the optoelectronic properties. The channel between neighboring electrodes had a width of 0.2 mm and a length of 1 mm. The width of the polarization light was ∼0.15 mm. During the experiment, the excitation light was not shined on electrodes. Current vs. voltage (*I–V*), photocurrent vs. time (*I–t*) and voltage vs. time (*V–t*) with light on and off were measured using a high precision electrometer (Keithley 6517B) and collected by continuous-wave lasers (ITC4001). The temperature during measurements was controlled using a Linkam TS1500 heating stage. The time-dependent photocurrent response was recorded by a current meter and an oscilloscope after switching the illumination.

### Determination of exciton binding energy (*E_B_*)

It is assumed that the photogenerated exciton depopulation is mainly dominated by radiative spontaneous emission processes and thermal dissociation. Therefore, at higher temperature, the decrease in photoluminescence intensity will solely originate from the increase in exciton thermal dissociation rate. The integrated photoluminescence intensity as a function of temperature can be fitted by Arrhenius Equation:

$$\begin{equation*}
I \!\left(T \right) = \ \frac{{{I_0}}}{{1 + A{e^{\left( { - Eb/{K_B}T} \right)}}}},
\end{equation*}$$

in which *K_B_* is Boltzmann constant and *I*_0_ represents the low-temperature integrated photoluminescence intensity.

### Material characterization

The single-crystal x-ray diffraction was performed on a Bruker D8 diffractometer with Mo Kα radiation. PXRD measurements were conducted on a Rigaku MiniFlex II diffractometer. Scanning electron microscopy images were collected on an SU 8010 field-emission scanning electron microscope, which was operated at 3 kV. The absorption spectra of MAPbBr_3_, (4-AMP)(MA)_2_Pb_3_Br_10_, and heterostructure were measured on the Perkin-Elmer Lambda 900 UV–Vis–NIR spectra photometer. The emission decay dynamics were monitored by FLS980’s single-photon counting capability (1024 channels; 1 μs window). For temperature-dependent photoluminescence emission measurements, the samples were kept in a liquid nitrogen cryostat, and the temperature was collected from 310 to 77 K using a temperature controller.

## Supplementary Material

nwab044_Supplemental_Files
