# Supplementary material for: Rational design of high-quality 2D/3D perovskite heterostructure crystals for record-performance polarization-sensitive photodetection
Source: Natl Sci Rev. 2021 Mar 16;8(10):nwab044. doi: 10.1093/nsr/nwab044 (PMC8566186; doi:10.1093/nsr/nwab044)
Supplement: nwab044_Supplemental_Files [file nwab044_supplemental_files.zip › Supplementary_data.docx]

Supplementary for

**Rational design of high-quality 2D/3D perovskite heterostructure crystals for record-performance polarization-sensitive photodetection**

*Xinyuan Zhang,^1,^**^3^ Lina Li,^1,2^ Chengmin Ji,^1,2^ Xitao Liu,^1,2^ Qing Li,^3,4^ Kun Zhang,^5^ Yu Peng,^1^ Maochun Hong,^1,2^ and Junhua Luo^*,1,2^*

*^1^State Key Laboratory of Structural Chemistry, Fujian Institute of Research on the Structure of Matter, Chinese Academy of Sciences, Fuzhou, Fujian 350002, China*

*^2^Fujian Science & Technology Innovation Laboratory for Optoelectronic Information of China, Fuzhou, Fujian 350108, P. R. China*

*^3^University of Chinese Academy of Sciences, Beijing 100049, China*

*^4^Hangzhou Institute for Advanced Study, University of Chinese Academy of Sciences, Hangzhou 310024, China.*

*^5^School of Physical Science and Technology, ShanghaiTech University, Shanghai 201210, China.*

*^*^Correspondence author E-mail: jhluo@fjirsm.ac.cn*

**Supplementary Discussion**

**Supplementary Discussion 1:** Temperature-cooling method for the growth of 2D/3D single-crystalline heterostructure.

Firstly, solubilities of (4-AMP)(MA)_2_Pb_3_Br_10_ and MAPbBr_3_ were measured in HBr solvent as a function of temperature in the range of 20–105℃. The powder of (4-AMP)(MA)_2_Pb_3_Br_10_ (or MAPbBr_3_) were pre-prepared and slowly added into 20 ml heated HBr solvent until the powder was insoluble. Temperature of the HBr solvent was precisely controlled by a hot plate. During the process, mass of the (4-AMP)(MA)_2_Pb_3_Br_10_ (or MAPbBr_3_) powder added to HBr solvent was accurately weighed and recorded. Thus, the solubility (*S*) of (4-AMP)(MA)_2_Pb_3_Br_10_ (or MAPbBr_3_) at a certain temperature could be calculated by the Equation: *S* = *m*/(*M·V_HBr_*), where *m* is the mass of (4-AMP)(MA)_2_Pb_3_Br_10_ (or MAPbBr_3_) powder added to HBr solvent, *M* is the molar mass of corresponding 4-AMP)(MA)_2_Pb_3_Br_10_ (or MAPbBr_3_), and *V_HBr_* is volume of HBr solvent.

As shown in Supplementary Fig. 2A, the solubilities of both 2D and 3D perovskites in HBr solution increased upon heating, and at any fixed temperature, the solubility of (4-AMP)(MA)_2_Pb_3_Br_10_ was always significantly lower than that of MAPbBr_3_. We note that the key for successful 2D/3D heterostructure crystallization was to take advantages of this solubility discrepancy between 2D (4-AMP)(MA)_2_Pb_3_Br_10_ and 3D MAPbBr_3_ perovskites (please see our previous report for more details [[1](#_ENREF_1)]). The MAPbBr_3_ could epitaxially grow atop its 2D counterpart, despite large lattice mismatch between (4-AMP)(MA)_2_Pb_3_Br_10_ (*a* = 8.3 Å, *b* = 8.3 Å, *c* = 22.1 Å) and MAPbBr_3_ (*a* = *b* = *c* = 5.9 Å). This can be explained by the flexible nature of hybrid perovskites, which accommodated the lattice mismatch and enabled a quick sharp transition from 2D perovskite to 3D perovskite at interface. Previous studies found that the Young’s modulus of hybrid perovskites was at least one order of magnitude smaller than those of traditional inorganic semiconductors, for example GaAs, GaN and Si [[2](#_ENREF_2), [3](#_ENREF_3)]. Lower energy was required to create a dislocation to relax the lattice mismatch strain, so the strain of the lattice-mismatched materials could be accommodated by a layer of soft organic spacer.

Moreover, we found that precisely tuning the reaction conditions could optimize the thickness of both 2D and 3D layers. In detail, for the 2D layer, lower (4-AMP)Br_2_ concentration and lower initial reaction temperature could efficiently reduce its thickness, while maintaining the thickness of 3D layer. Experimental details were as follows:

| Conditions  2D  Thickness | MABr: (4-AMP)Br_2_:  (mmol) | HBr  (47%, ml) | Initial  Temp  (°C) | Final  Temp  (°C) | Cooling  Rate  (°C/day) |
| --- | --- | --- | --- | --- | --- |
| 1 mm | 3:1 | 100 | 80 | 35 | 0.8 |
| 0.8 mm | 4:1 | 100 | 70 | 35 | 0.8 |
| 0.6 mm | 4.5:1 | 100 | 65 | 35 | 0.8 |
| 0.2 mm | 5:1 | 100 | 50 | 35 | 0.8 |

On the other hand, a lower final reaction temperature could lead to thicker 3D phase. Experimental details were listed following.

| Conditions  3D  Thickness | MABr: (4-AMP)Br_2_:  (mmol) | HBr  (47%, ml) | Initial  Temp  (°C) | Final  Temp  (°C) | Cooling  Rate  (°C/day) |
| --- | --- | --- | --- | --- | --- |
| 0.18 mm | 4:1 | 100 | 70 | 40 | 0.8 |
| 0.30 mm | 4:1 | 100 | 70 | 35 | 0.8 |
| 0.35 mm | 4:1 | 100 | 70 | 30 | 0.8 |
| 0.42 mm | 4:1 | 100 | 70 | 23 | 0.8 |

Pictures of the grown heterostructure crystals with different 3D layer thickness were shown in Supplementary Fig. 2C and the corresponding MAPbBr_3_ growth rate was shown in Supplementary Fig. 2D. Furthermore, by partially substituting Pb^2+^ with Sn^2+^, we could also prepare Sn-based heterostructure crystals following this temperature-cooling solution-epitaxial method (Supplementary Fig. 2E showed the pictures of the grown (4-AMP)(MA)_2_Sn_1.8_Pb_1.2_Br_10_/MASn_0.6_Pb_0.4_Br_3_ heterostructure crystals). The method for the heterostructure growth we come up with may hold potential broad applicability to other hybrid perovskites with varied structures and prospectively, can be extended to other material systems, opening up the opportunities for a host of high-performance optoelectronic devices.

Here we are sharing our recipes for the growth of (4-AMP)(MA)_2_Pb_3_Br_10_/MAPbBr_3_ heterostructure crystal used in the experiment as a reference: stoichiometric MABr (methylammonium bromidum, 8 mmol, 0.90 g) and PbBr_2_ (10 mmol, 3.67 g) were slowly dissolved in the aqueous HBr solution (100 mL, 48%) by heating to boiling, which formed a bright-yellow solution. Subsequent addition of (4-AMP)Br_2_ (4-(aminomethyl)piperidinium bromidum, 2 mmol, 0.54 g) into the solution initially yielded the yellow powder precipitation. After re-dissolution by heating, a clear-yellow solution was obtained. Then, the prepared solution was placed on a hot plate with initial temperature as 70°C and subjected to a controlled cooling rate of 0.8°C/day. After cooling the solution to 35°C, large-sized heterostructure crystals could be obtained.

**Supplementary Discussion 2:** The single-crystalline quality and interfacial quality of the as-grown heterostructure crystal.

Phase purity of the (4-AMP)(MA)_2_Pb_3_Br_10_/MAPbBr_3_ heterostructure was confirmed by powder x-ray diffraction (PXRD, Supplementary Fig. 3A) and energy-dispersive x-ray spectroscopy (EDS, Supplementary Fig. 3B). Single crystal x-ray diffraction (SCXRD) patterns of (4-AMP)(MA)_2_Pb_3_Br_10_/MAPbBr_3_ heterostructure at (001) plane consisted lattice information from (4-AMP)(MA)_2_Pb_3_Br_10_ and MAPbBr_3_ crystals (Supplementary Fig. 4A−C), which confirmed the formation of heterostructure and indicated its high single crystalline quality. Scanning electron microscopy (SEM) studies in Supplementary Fig. 4D proved the high crystalline quality with few surface defects, which was consistent with atomic force microscopy (AFM, Supplementary Fig. 4E) characterization. Interfacial morphology revealed by SEM image supported a near-atomically sharp and intimate interface (Supplementary Fig. 4F). For practical applications, such an interface is highly desirable because it will facilitate the formation of built-in electrical field, and thus the optoelectronic devices. To further evaluate the crystalline quality, we quantitatively measured the trap density (*N*_trap_) of heterostructure crystals as well as the (4-AMP)(MA)_2_Pb_3_Br_10_ crystals through space-charge-limited current (SCLC) technique (Supplementary Fig. 5). The single crystal devices were fabricated with a structure of Au/single crystal/Au for hole-only devices. The trap density was calculated according the calculation based on follow equation:

$$N_{trap}= \frac{2\varepsilon\varepsilon_{0}V_{TFL}}{eL^{2}}$$

where *ε* is relative dielectric constant, *ε*_0_ is vacuum permittivity, *V*_TFL_ is the onset voltage of the trap-flled limit region, *L* is the thickness of the material and *e* presents the element charge. The *N*_trap_ of the heterostructure crystal was determined as low as 5.2 × 10^10^ cm^-3^, which was comparable to the (4-AMP)(MA)_2_Pb_3_Br_10_ crystal (2.8 × 10^10^ cm^-3^), indicating similar crystallinity. The value of 5.2 × 10^10^ cm^-3^ was also comparable to the state-of-art MAPbX_3_ (X = Cl, Br, I) single crystals (*N*_trap_ = 3 × 10^10^ cm^-3^) [[4](#_ENREF_4), [5](#_ENREF_5)]. Moreover, we fitted the current–voltage curve for the bias voltage > 15 V to calculate the hole mobility using the Mott’s SCLC theory [[6](#_ENREF_6)].

$$J_{D}= \frac{9\varepsilon\varepsilon_{0}\mu V_{b}^{2}}{8L^{3}}$$

where *J_D_, ε, ε_0_, µ, V_b_,* and *L* are the dark current density, the relative dielectric constant, the vacuum permittivity, the mobility of crystal, applied voltage, and the thickness of the crystal, respectively. Interestingly, the hole mobility *µ* in the heterostructure crystal was about 6 cm^2^ V^-1^ s^-1^, which was more than 10 times larger than its 2D counterpart (4-AMP)(MA)_2_Pb_3_Br_10_ (0.5 cm^2^ V^-1^ s^-1^), demonstrating the significantly improved carrier mobility after the incorporation of 3D perovdskite.

**Supplementary Figures**

**
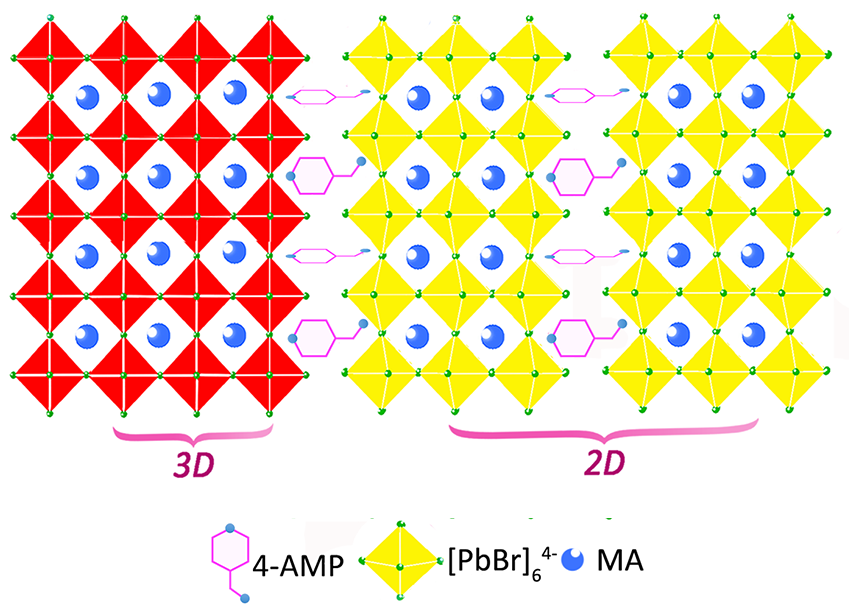
**

**Supplementary Figure 1.** Schematic illustration of the 2D/3D heterostructure.

**
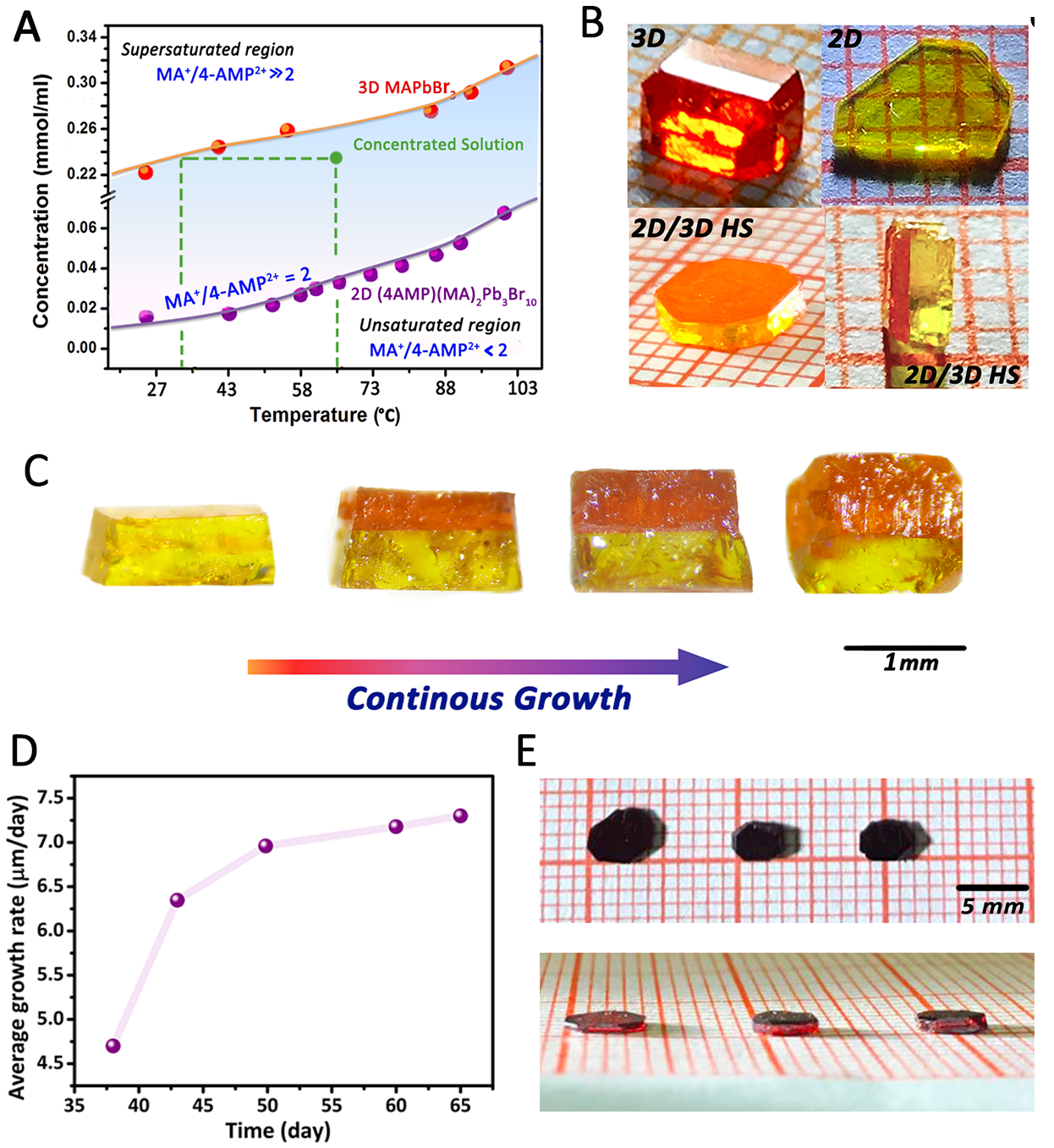
**

**Supplementary Figure 2.** Growth of 2D/3D heterostructure crystals. (A) Solubility curves of (4-AMP)(MA)_2_Pb_3_Br_10_ and MAPbBr_3_, respectively. (B) Pictures of the grown (4-AMP)(MA)_2_Pb_3_Br_10_, MAPbBr_3_, and heterostructure crystals. (C) (4-AMP)(MA)_2_Pb_3_Br_10_/MAPbBr_3_ heterostructure crystals with different thickness of 3D layer. (D) The growth rate of the MAPbBr_3_ layer. (E) Pictures of the grown (4-AMP)(MA)_2_Sn_1.8_Pb_1.2_Br_10_/MASn_0.6_Pb_0.4_Br_3_ heterostructure crystals with different thickness.


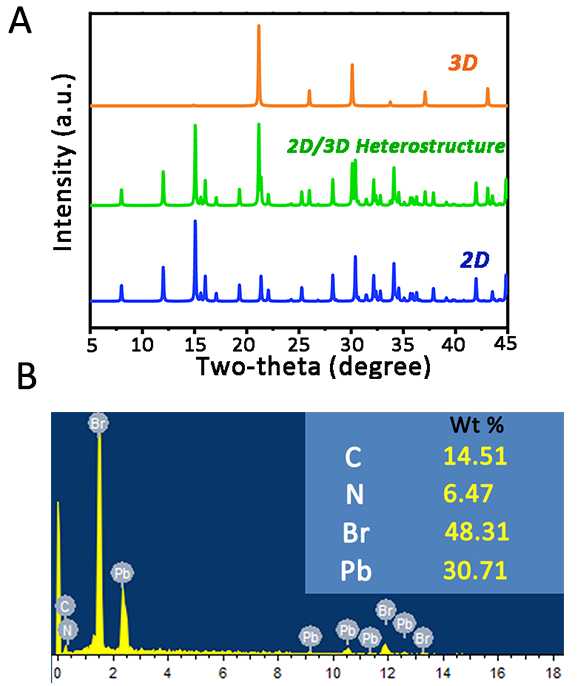


**Supplementary Figure 3.** Phase purity of the as-grown heterostructure. (A) PXRD patterns of 2D (4-AMP)(MA)_2_Pb_3_Br_10_, 3D MAPbBr_3_, and the 2D/3D heterostructure, respectively. (B) EDS of the 2D/3D heterostructure.


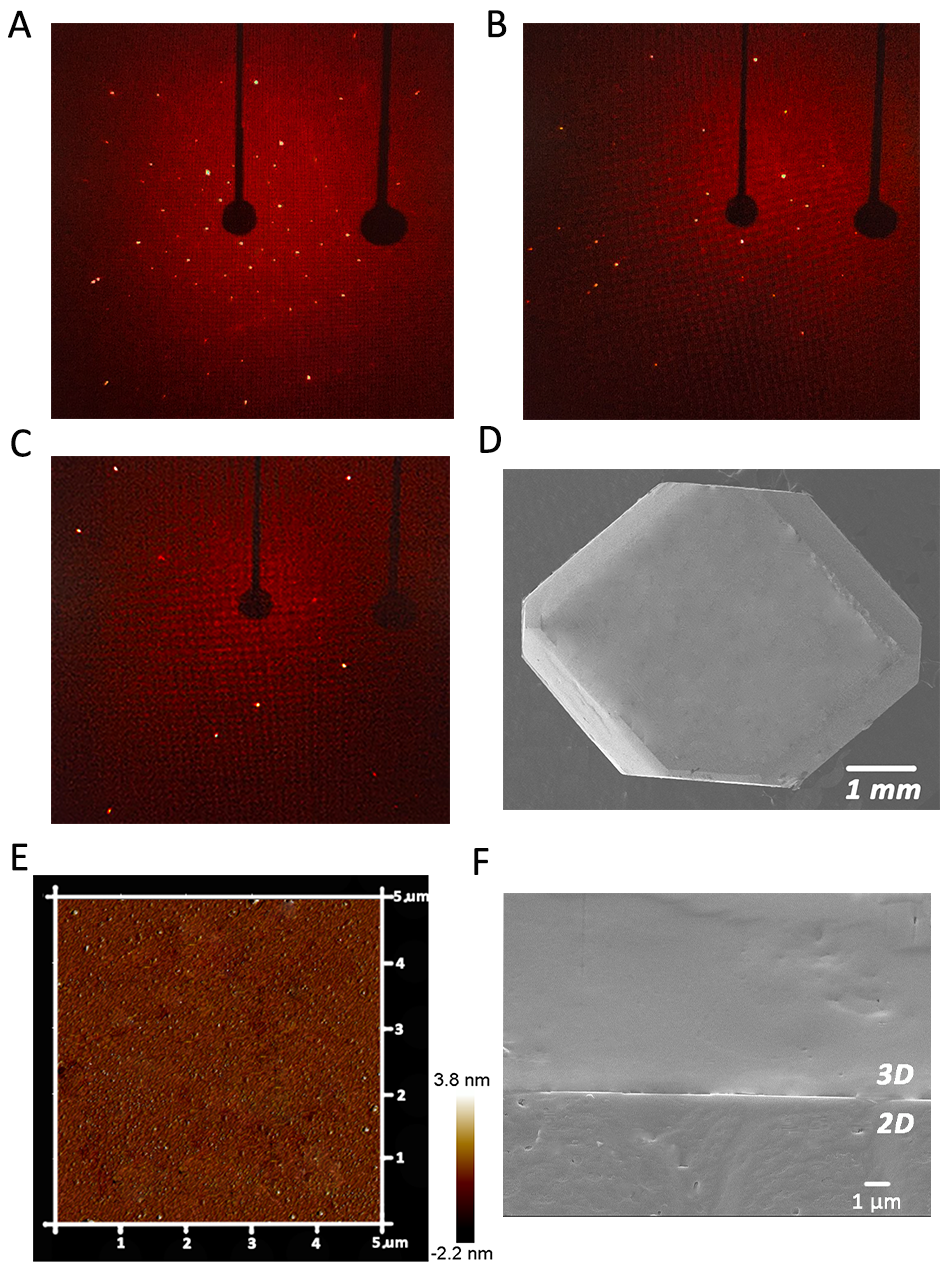


**Supplementary Figure 4.** Characterizations of 2D/3D heterostructure crystal. SCXRD patterns of (A) the heterostructure crystal, (B) (4-AMP)(MA)_2_Pb_3_Br_10_ and (C) MAPbBr_3_ crystals patterns at (001) plane, respectively. (D) SEM image of the heterostructure crystal. (E) AFM image of epitaxial 3D MAPbBr_3_. (F) SEM image at the heterostructure interface.


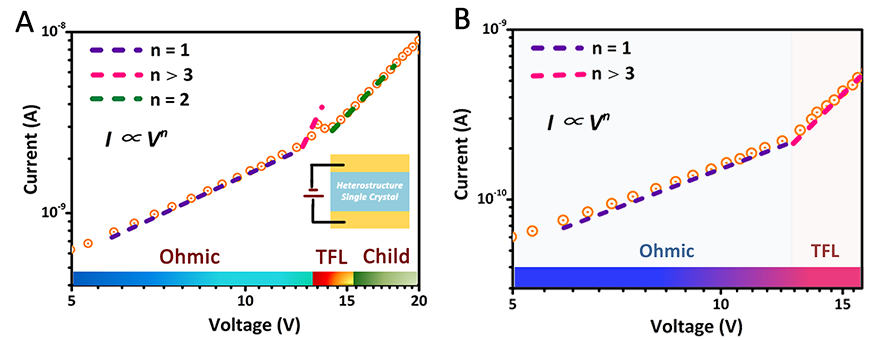


**Supplementary Figure 5.** Trap density measurements. Trap densities of (A) the 2D/3D heterostructure. (B) 2D perovskite measured through SCLC method.


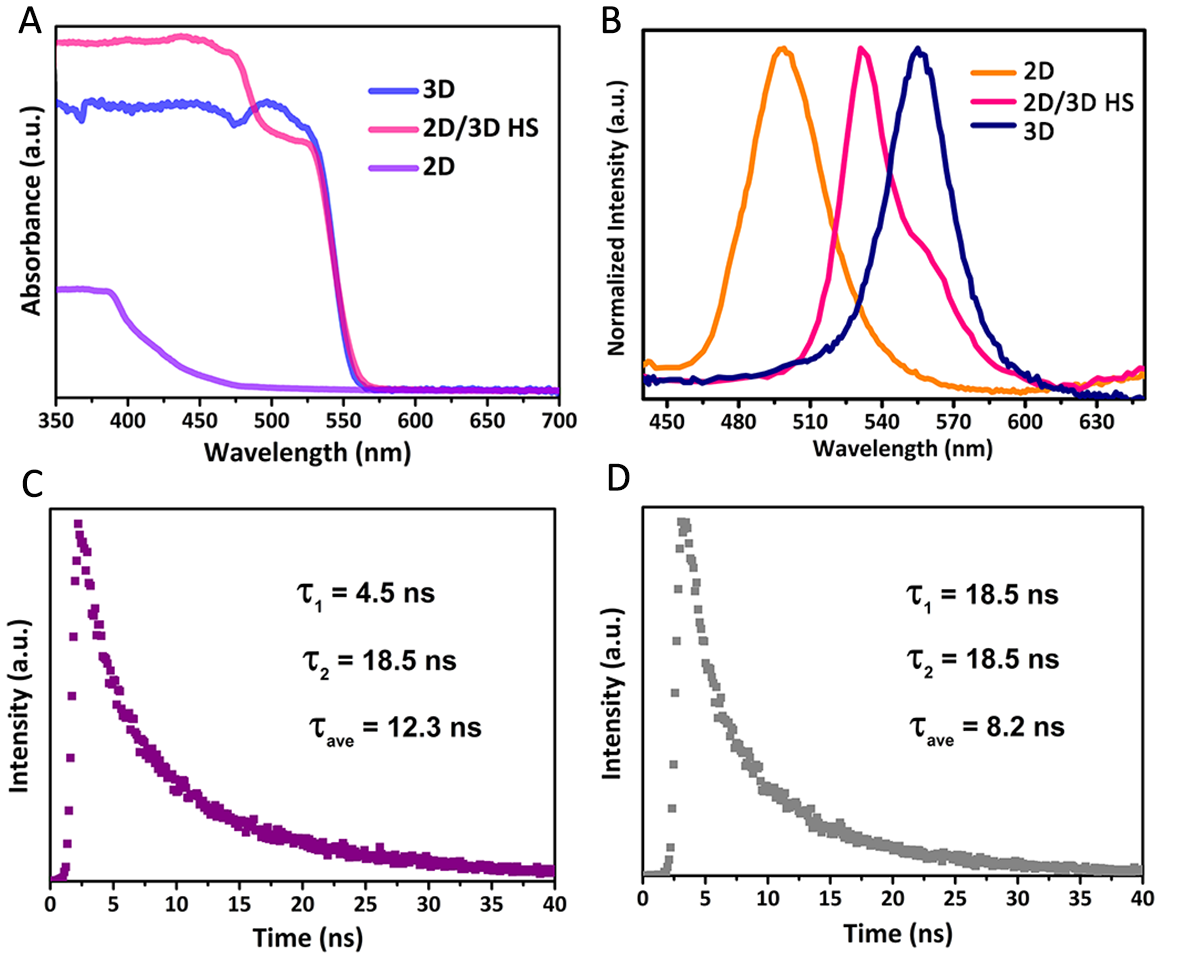


**Supplementary Figure 6.** Optical properties of the heterostructures. (A) Absorption spectra of the 2D (4-AMP)(MA)_2_Pb_3_Br_10_, 3D MAPbBr_3_, and the 2D/3D heterostructure. (B) PL spectra of the 2D (4-AMP)(MA)_2_Pb_3_Br_10_, 3D MAPbBr_3_, and the 2D/3D heterostructure. PL lifetime of the 2D/3D heterostructure at (C) 560 nm and (D) 540 nm.


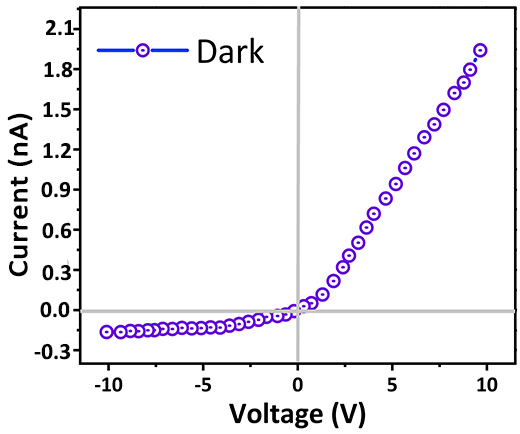


**Supplementary Figure 7.** *I–V* curves of the heterostructure device measured in the dark.


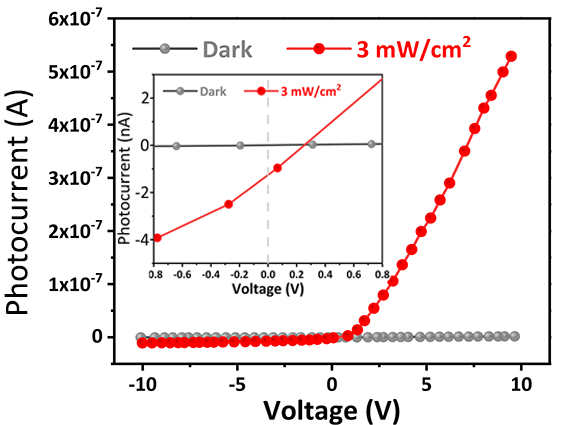


**Supplementary Figure 8.** *I–V* curves of the heterostructure device measured under 405 nm laser illumination. The insertion shows an obvious photovoltaic effect with an open-circuit voltage of ∼0.25 V and short-circuit current of ∼1.5 nA.


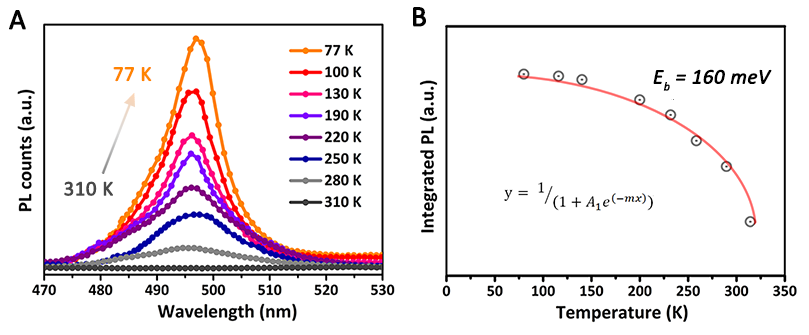


**Supplementary Figure 9.** PL spectra of 2D perovskite. (A) Temperature-dependent PL spectra of (4-AMP)(MA)_2_Pb_3_Br_10_. (B) Exciton binding energy (*E*_b_) determination of 2D (4-AMP)(MA)_2_Pb_3_Br_10._


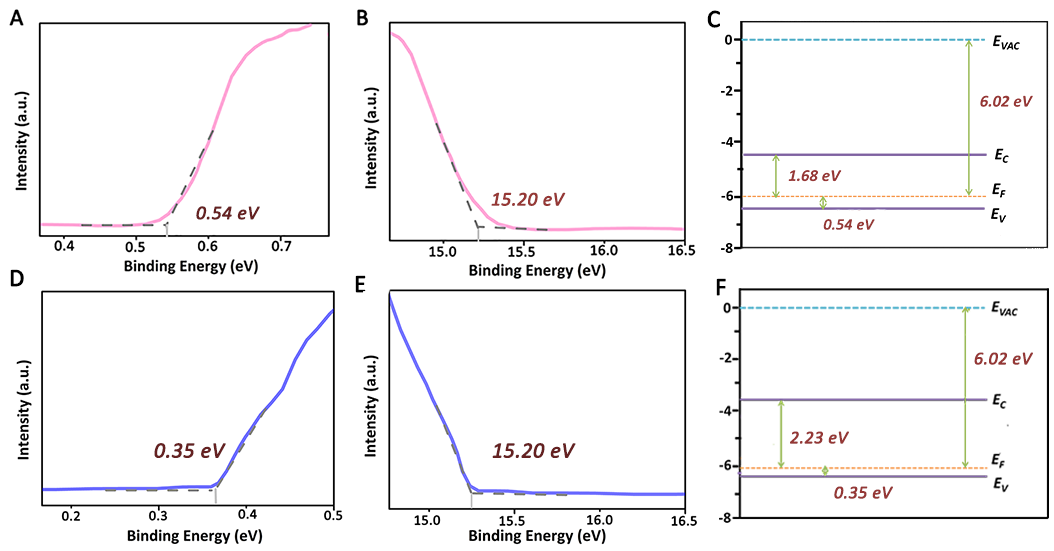


Supplementary Figure 10. Ultra-violet photoelectron spectroscopy (UPS) for 2D and 3D perovskites. (A) and (B) The UPS measurement of 3D MAPbBr_3_. (C) Schematic evolution of Fermi level position and bandgap edge derived from UPS for MAPbBr_3_. (D) and (E) The UPS measurement of 2D (4-AMP)(MA)_2_Pb_3_Br_10_. (F) Schematic evolution of Fermi level position and bandgap edge derived from UPS for (4-AMP)(MA)_2_Pb_3_Br_10_. *E_VAC_* is [vacuum](javascript:;) [level](javascript:;)；*E_C_* is conduction band [level](javascript:;); *E_F_* is Fermi level and *E_V_* is valence band [level](javascript:;). UPS was used to determine the valence band minima (VBM). Conduction band minima (CBM) were calculated using the optical band gaps.


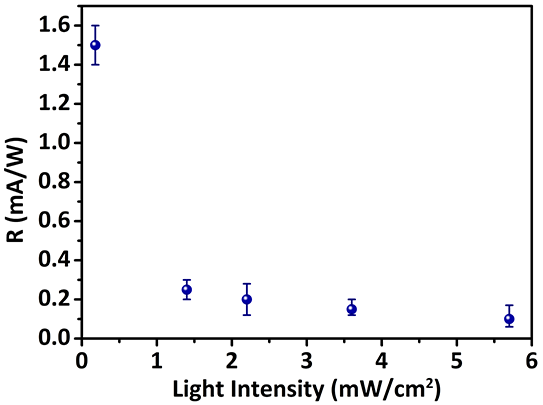


Supplementary Figure 11. Incident-light power dependence of photoresponsivity of the heterostructure._._


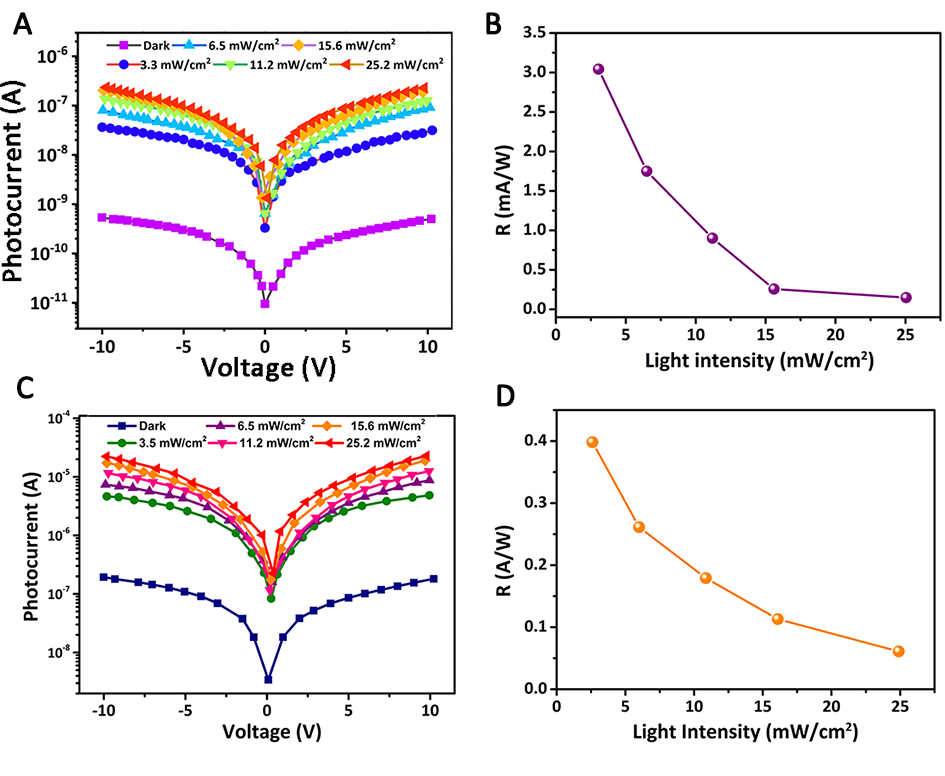


Supplementary Figure 12. Device performance of 2D and 3D perovskites. (A) *I–V* curves of the photodetector based on 2D (4-AMP)(MA)_2_Pb_3_Br_10_ crystal measured under 405 nm laser illumination. (B)_._ Incident-light power dependence of photoresponsivity of the 2D perovskite-based device. (C) *I–V* curves of the photodetector based on 3D MAPbBr_3_ crystal measured under 520 nm laser illumination. (D) Incident-light power dependence of photoresponsivity of the device based on MAPbBr_3_.


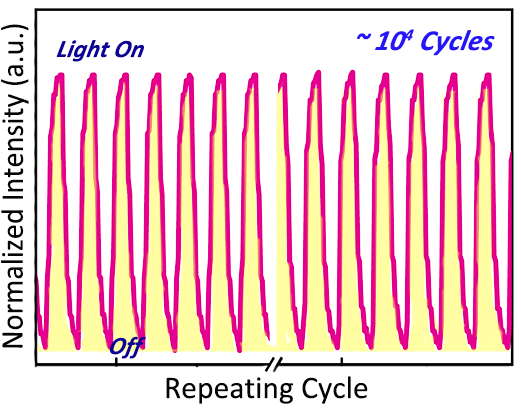


Supplementary Figure 13. Transient photocurrent response of the photodetector based on heterostructure crystal.


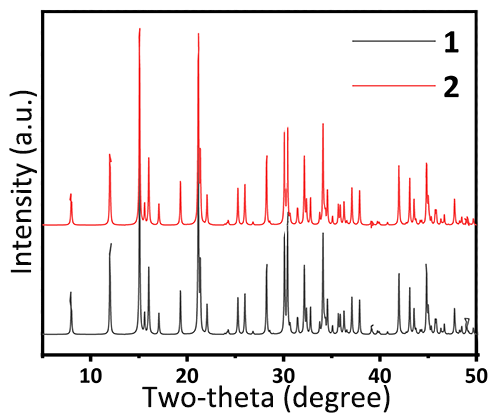


Supplementary Figure 14. PXRD patterns of the heterostructure detector after storage in air for 30 days. Line 1: fresh; Line 2: after 30-days storage.

Supplementary Tables

Supplementary Table 1 Performance comparison of (4-AMP)(MA)_2_Pb_3_Br_10_/MAPbBr_3_ heterostructure polarization-sensitive photodetector with other devices.

|  | **Devices** | **λ (nm)** | **on/off radio** | **Polarization**  **Radio** | **D* (Jones)** | **Speed**  **(μs)** | **Condition** | **Ref** |
| --- | --- | --- | --- | --- | --- | --- | --- | --- |
| **Single-Phase two dimensi-onal material** | **(AA)_2_(EA)_2_Pb_3_Br_10_** | **405** | **~10^4^** | **~15** | **2.5×10^9^** |  | **Self-driven**  **ferroelectric** | **[7]** |
|  | **(BA)_2_MAPb_2_Br_7_** | **405** | **~10^3^** | **~2** | **1.1×10^9^** | **2.0/2.8** | **10V** | **[8]** |
|  | **(i-PA)_2_CsAgBiBr_7_** | **405** | **~10^3^** | **~1.35** |  | **200/400** | **10V** | **[9]** |
|  | **GeSe** | **532** |  | **~1.09** |  |  | **2V** | **[10]** |
|  | **GeAs_2_** | **532** |  | **~2** |  |  | **1V** | **[11]** |
|  | **BP** | **1200** |  | **~3.5** |  |  | **0.1V** | **[12]** |
| **Nanow-**  **ire** | **CsPbBr_3_** | **470** | **~10^3^** | **~2.6** |  | **21/23** | **5V** | **[13]** |
|  | **MAPbI_3_** | **530** | **~10^3^** | **~1.3** | **2×10^13^** | **100** | **1V** | **[14]** |
|  | **InP** | **514** |  | **~10** |  |  | **0.05V** | **[15]** |
| **Heteros-**  **tructure** | **Graphene/PdSe_2_/Ge** | **650** | **~10^5^** | **~112** | **~10^13^** | **6.4/92.5** | **Self-driven** | **[16]** |
|  | **SbI_3_/SbO_3_** | **450** | **~10^2^** | **~3** | **~2×10^9^** | **~2×10^5^** | **8V** | **[17]** |
|  | **BP/hBN** | **mid-IR** |  | **~6.4** |  |  |  | **[18]** |
|  | **hBN/(b-As_x_P_1-x_)/hBN** | **5000** |  | **~14** | **~2×10^10^** |  | **1V** | **[19]** |
|  | **BP/InSe** | **633** | **~10^3^** | **~10.76** |  | **24000/**  **32000** | **Self-driven** | **[20]** |
|  | **BPt/MoS_2_/BPb** | **3500** |  | **~22** | **~10^10^** |  |  | **[21]** |
|  | **PdSe_2_/FA_1-x_Cs_X_PbI_3_** | **808** | **~10^4^** | **~6** | **~10^13^** | **3.5/4** | **Self-driven** | **[22]** |
|  | **(4-AMP)(MA)_2_Pb_3_Br_10_**  **/MAPbBr_3_** | **405** | **~10^5^** | **~17** | **~10^12^** | **600/600** | **Self-driven** | **This work** |

AA = allyammonium; EA = ethylammonium; i-PA = isopentylammonium; BA = butylammonium.

Supplementary Table 2 Structural comparison of (4-AMP)(MA)_2_Pb_3_Br_10_ and MAPbBr_3_.

| Empirical formula | CH_3_NH_3_PbBr_3_ | (4-AMP)(MA)_2_Pb_3_Br_10_ |
| --- | --- | --- |
| Formula weight(g/mol) | 478.97 | 1608.8 |
| Measurement temperature/K | 296 | 135 |
| Crystal habit | Orange | Yellow |
| Crystal system | Cubic | Orthorhombic |
| Space group | *Pm-3m* | *P*ba2 |
| a/Å | 5.9171 | 8.3087 |
| b/Å | 5.9171 | 8.3087 |
| c/Å | 5.9171 | 22.104 |
| α/° | 90.0 | 90.0 |
| β/° | 90.0 | 90.0 |
| γ/° | 90.0 | 90.0 |
| Volume/Å^3^ | 207.2 | 1526.0 |
| Z | 1 | 2 |
| Density/g·cm^-3^ | 3.925 | 3.484 |

**Supplementary References**

[1] Zhang X, Ji C and Liu X *et al.* Solution-Grown Large-Sized Single-Crystalline 2D/3D Perovskite Heterostructure for Self-Powered Photodetection. *Adv. Opt. Mater.* 2020; **8**: 2000311.

[2] Wei W, Zhang Y and Xu Q *et al.* Monolithic integration of hybrid perovskite single crystals with heterogenous substrate for highly sensitive X-ray imaging. *Nat. Photon.* 2017; **11**: 315−21.

[3] Wang J, Huang Q and Yu H. Size and temperature dependence of Young's modulus of a silicon nano-plate. *J. Phys. D* 2008; **41**: 165406.

[4] Shi D, Adinolfi V and Comin R *et al.* Low trap-state density and long carrier diffusion in organolead trihalide perovskite single crystals. *Science* 2015; **347**: 519−2.

[5] Dong Q, Fang Y and Shao Y *et al.* Electron-hole diffusion lengths > 175 mm in solution grown CH_3_NH_3_PbI_3_ single crystals. *Science* 2015; **347**: 967−70.

[6] Liu Y, Zhang Y and Zhao K *et al.* A 1300 mm^2^ Ultrahigh-Performance Digital Imaging Assembly using High-Quality Perovskite Single Crystals. *Adv. Mater.* 2018; **30:** 1707314.

[7] Peng Y, Liu X and Sun Z et al. Exploiting Bulk Photovoltaic Effect in a 2D Trilayered Hybrid Ferroelectric for Highly Sensitive Polarized Light Detection. *Angew. Chem. Int. Ed.* 2020; **59**: 3933–37.

[8] Li L, Liu X and Li Y *et al.* Two-dimensional hybrid perovskite-type ferroelectric for highly polarization-sensitive shortwave photodetection. *J. Am. Chem. Soc.* 2019; **141**: 2623−9.

[9] Li Y, Yang T and Xu Z *et al.* Dimensional reduction of Cs_2_AgBiBr_6_: 2D hybrid double perovskite with strong polarization-sensitivity. *Angew. Chem. Int. Ed.* 2020; ***59***: 3429–33.

[10] Wang X, Li Y and Huang L *et al.* Short-wave near-infrared linear dichroism of two dimensional germanium selenide. *J. Am. Chem. Soc.* 2017; ***139***: 14976–82.

[11] Li L, Gong P and Sheng D *et al.* Highly in-plane anisotropic 2D GeAs_2_ for polarization sensitive photodetection. *Adv. Mater.* 2018; ***30***: e1804541.

[12] Yuan H, Liu X and Afshinmanesh F *et al.* Polarization-sensitive broadband photodetector using a black phosphorus vertical p-n junction. *Nat. Nanotech.* 2015; ***10***: 707–13

[13] Feng J, Yan X and Liu Y *et al.* Crystallographically aligned perovskite structures for highperformance polarization-sensitive photodetectors. *Adv. Mater.* 2017; ***29***: 1605993

[14] Gao L, Zeng K and Guo J *et al.* Passivated single-crystalline CH_3_NH_3_PbI_3_ nanowire photodetector with high detectivity and polarization sensitivity. *Nano Lett.* 2016; ***16***: 7446–54.

[15] Wang J, Gudiksen M and Duan X *et al.* Highly polarized photoluminescence and photodetection from single indium phosphide nanowires. *Science* 2001; ***293***: 1455–57.

[16] Wu D, Guo J and Du J *et al.* Highly polarization-sensitive, broadband, self-powered photodetector based on Graphene/PdSe_2_/Germanium heterojunction. *ACS Nano* 2019; ***13***: 9907–17.

[17] Xiao M, Yang H and Shen W. *et al.* Symmetry-reduction enhanced polarization-sensitive photodetection in core-shell SbI_3_/Sb_2_O_3_ van der Waals heterostructure. *Small* 2020; ***16***: e1907172

[18] Chen X, Lu X and Deng B *et al.* Widely tunable black phosphorus mid-infrared photodetector. *Nat. Commun.* 2017; ***8***: 1672.

[19] Yuan S, Shen C and Deng B *et al.* Air-stable room-temperature mid-infrared photodetectors based on hBN/Black Arsenic Phosphorus/hBN heterostructures. *Nano lett.* 2018; *18*: 3172–9..

[20] Zhao S, Wu J and Jin K *et al.* Highly polarized and fast photoresponse of black phosphorus InSe vertical p-n heterojunctions. *Adv. Funct. Mater.* 2018; ***28***: 1802011.

[21] Bullock J, Amani M and Cho J *et al.* A. Polarization-resolved black phosphorus/molybdenum disulfide mid-wave infrared photodiodes with high detectivity at room temperature. *Nat. Photon.* 2018; ***12***: 601–7.

[22] Zeng L, Chen Q and Zhang Z *et al.* Multilayered PdSe_2_/Perovskite schottky junction for fast, self-powered, polarization-sensitive, broadband photodetectors, and image sensor application. *Adv. Sci.* 2019; **6**: 1901134.
